# Prioritization of gene regulatory interactions from large-scale modules in yeast

**DOI:** 10.1186/1471-2105-9-32

**Published:** 2008-01-22

**Authors:** Ho-Joon Lee, Thomas Manke, Ricardo Bringas, Martin Vingron

**Affiliations:** 1 Department of Computational Molecular Biology, Max Planck Institute for Molecular Genetics, 14195 Berlin, Germany; 2 Centro de Ingenieria Genetica y Biotecnolgia, Cubanacan, CP 6162, La Habana, Cuba

## Abstract

**Background:**

The identification of groups of co-regulated genes and their transcription factors, called transcriptional modules, has been a focus of many studies about biological systems. While methods have been developed to derive numerous modules from genome-wide data, individual links between regulatory proteins and target genes still need experimental verification. In this work, we aim to prioritize regulator-target links within transcriptional modules based on three types of large-scale data sources.

**Results:**

Starting with putative transcriptional modules from ChIP-chip data, we first derive modules in which target genes show both expression and function coherence. The most reliable regulatory links between transcription factors and target genes are established by identifying intersection of target genes in coherent modules for each enriched functional category. Using a combination of genome-wide yeast data in normal growth conditions and two different reference datasets, we show that our method predicts regulatory interactions with significantly higher predictive power than ChIP-chip binding data alone. A comparison with results from other studies highlights that our approach provides a reliable and complementary set of regulatory interactions. Based on our results, we can also identify functionally interacting target genes, for instance, a group of co-regulated proteins related to cell wall synthesis. Furthermore, we report novel conserved binding sites of a glycoprotein-encoding gene, CIS3, regulated by Swi6-Swi4 and Ndd1-Fkh2-Mcm1 complexes.

**Conclusion:**

We provide a simple method to prioritize individual TF-gene interactions from large-scale transcriptional modules. In comparison with other published works, we predict a complementary set of regulatory interactions which yields a similar or higher prediction accuracy at the expense of sensitivity. Therefore, our method can serve as an alternative approach to prioritization for further experimental studies.

## Background

There have been many studies about groups of genes and their transcription factors (TFs), called transcriptional modules, by integrating heterogeneous data sources such as chromatin immunoprecipitation on microarray (ChIP-chip), gene expression data and cis-regulatory motifs [1-8]. ChIP-chip data alone do not possess functional regulatory information and gene expression data alone do not contain physical binding information. Data integration tries to compensate for such limitations of a single type of data source alone. This process can often generate more complete biological hypotheses than those from each data source separately. A particularly important issue in biology is to discriminate regulatory binding of TFs from mere physical binding events. One way to tackle this problem is to integrate functional data such as gene expression with physical binding data such as ChIP-chip [1,9].

Modules are believed to be a fundamental organizational unit of cellular networks [10,11]. Transcriptional modules are one type of such modules related to gene regulatory networks of TFs and target genes. The computational studies mentioned above aimed at the identification of such modules as independent or inter-connected functional units in regulatory networks. Experimentalists face the challenge to verify predicted modules in their functional contexts at the level of *all *individual links. This is currently impossible as the number of regulatory links in modules predicted from large-scale data analysis is in the order of thousands. In this work, we aim to prioritize regulatory interactions in transcriptional modules as an attempt to overcome this issue.

**Figure 1:**
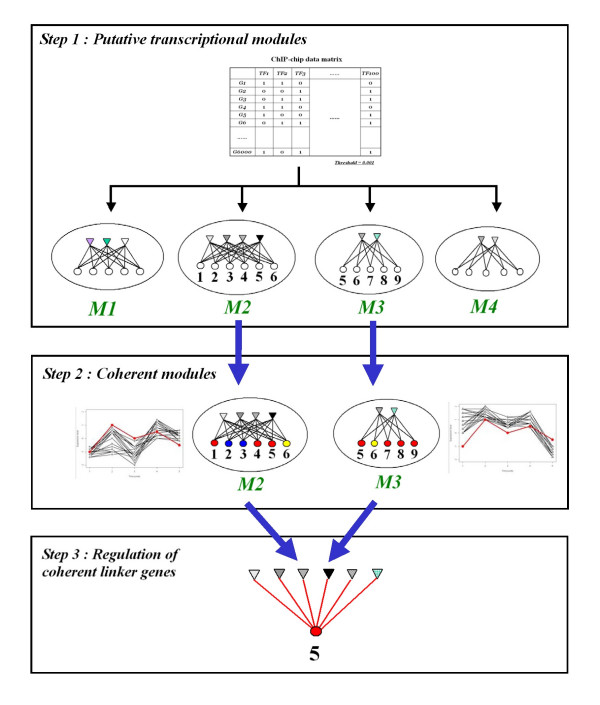
**Overview of our method**. In Step 1, we generate all possible putative transcriptional modules (PTMs) from ChIP-chip data as described in Methods section. Here we show a toy example of ChIP-chip binary data matrix and corresponding 4 PTMs, M1 to M4. Each module contains a set of transcription factors (triangles) and a set of target genes (circles) connected by links between all of them (bicliques; see Eq. (1)). The genes in M2 and M3 are numbered for an illustration purpose below. In Step 2, we identify *coherent modules *among all PTMs using gene expression and functional annotation data. Only those PTMs which satisfy Eq. (5) are selected as coherent modules. In the given example in the figure, M1 and M4 are meant to be non-coherent and hence discarded altogether. Colours of genes in both coherent modules, M2 and M3, symbolize different functions. 'Blue' and 'red' functions are meant to be coherent (enriched) in the respective modules. Notice that the 'red' function is coherent in both modules. The fictitious 'M'-shaped expression profiles are also shown to be coherent as well in both modules. The red profile belongs to gene 5 which is annotated to the coherent 'red' function. Finally, in Step 3, we identify those genes which appear in multiple coherent modules with any common coherent functions. This is illustrated in Step2 by the gene 5 which belong to *both *coherent modules. The gene is annotated to the *common *coherent 'red' function in both modules. The union of regulators in M2 and M3 is predicted to functionally regulate the gene in this illustration. We term such genes 'coherent linker genes'. Notice that gene 6 belonging to both modules is not a coherent linker gene because its annotated 'yellow' function is not coherent in the modules. See the text for more details.

Our approach starts with putative transcriptional modules (PTMs) derived from genome-wide ChIP-chip data (Step 1 in Figure 1). These PTMs are defined by all possible combinations (or subsets) of regulators and their respective bound genes at a given ChIP-chip p-value threshold [1,12]. Then our algorithm identifies a subset of PTMs which are (1) coherent in expression profiles of target genes and at the same time (2) enriched in functional categories (Step 2 in Figure 1). That is, both gene expression and functional annotation data are used to extract functional signals after binding signals are retrieved from ChIP-chip data. We use the terms, 'functional' and 'regulatory', interchangeably when we discuss TF binding throughout this study. All links between TFs and target genes in the identified subset of PTMs, called coherent modules, are considered candidate functional links. The goal is then to narrow down those candidate functional links to core functional links. Our key strategy is to focus on intersections of coherent modules for all enriched functional categories (Step 3 in Figure 1). This short list of TF-gene pairs is our predicted functional pairs and consequently has priority over the others in coherent modules for further mechanistic analysis or experimental validation.

Our method is applied to ChIP-chip data by Lee et al. [13], gene expression data by Spellman et al. [14], and MIPS functional category data [15]. We evaluate our method in terms of the number of true functional links between TFs and target genes among our predictions with respect to two reference datasets (one from literature and the other from conserved binding motifs). We also compare our method with two previous algorithms, GRAM [1] and MA-Networker [9], which integrated two of our data sources, ChIP-chip and gene expression data. Finally, we investigate our predictions in more detail and focus on those predicted TF-gene pairs whose expression profiles are highly correlated with each other. This further enables us to suggest functional interaction among gene products and novel conserved binding sites for those pairs.

## Results

### Putative transcriptional modules

We generated a total of 584 putative transcriptional modules (PTMs) by taking all non-empty subsets of 106 TFs from ChIP-chip data by Lee et al. with a binding p-value threshold 0.001 (see Methods; Step1 in Figure 1). We imposed a constraint of the minimum number of target genes in all modules being 5 for the purpose of statistical assessment in our subsequent analysis. The list of PTMs was examined in subsequent analysis by incorporating gene expression and functional annotation data.

### Prioritization of TF-gene functional links from coherent modules

**Figure 2:**
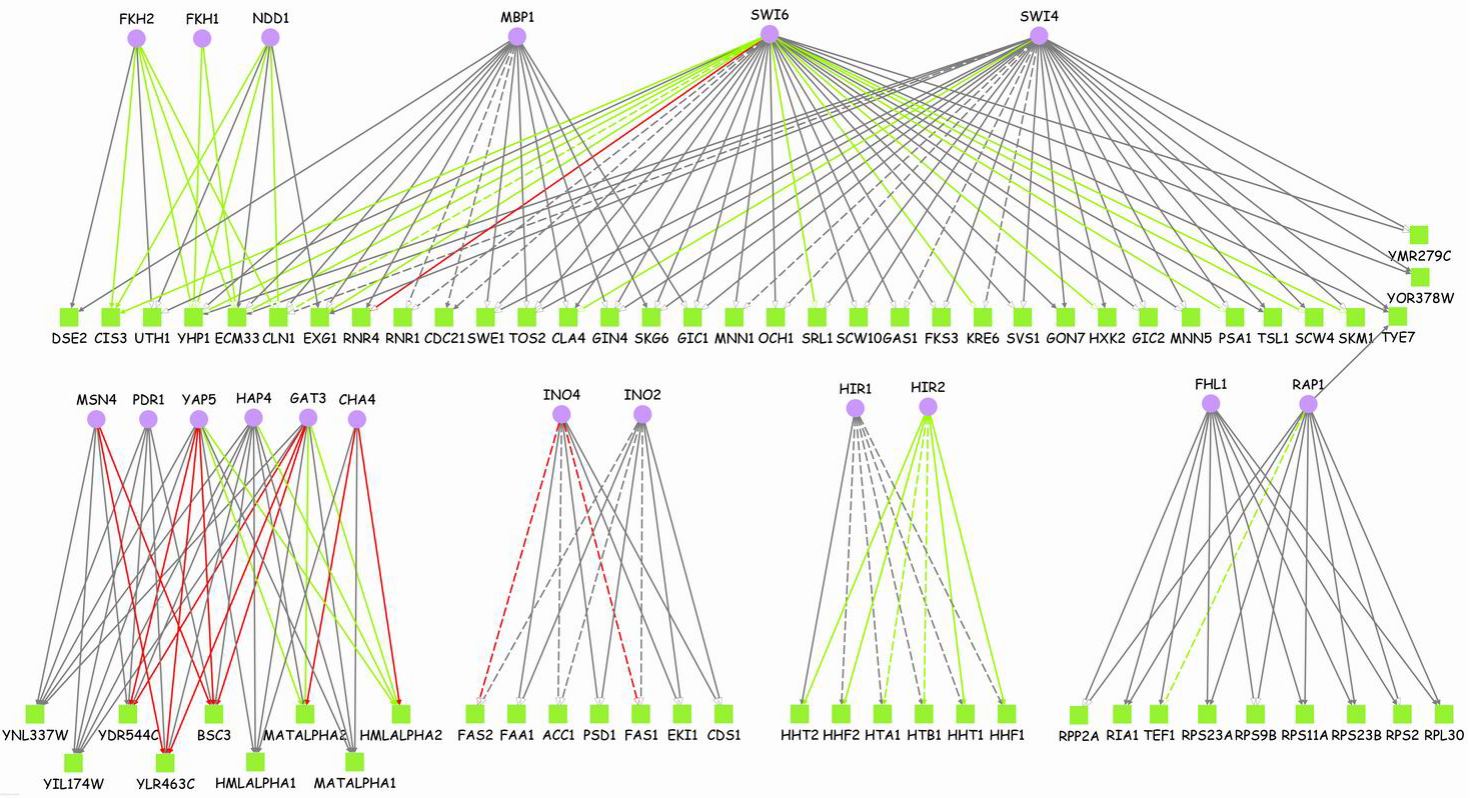
**Summary of our final predictions**. This network diagram is a graphical representation of our final predicted 177 TF-gene functional pairs between 18 TFs and 66 target genes (coherent linker genes). Dashed links show 24 literature-verified pairs. Links with white arrow heads represent 85 pairs with conserved motifs. Green and red links show additional information about expression correlation between TF-encoding genes and their target genes: high positive (Pearson coefficients > 0.661) and negative correlation (Pearson coefficients < -0.628) respectively. For example, the predicted functional pair of Rap1 and TEF1 (on the bottom right) is confirmed with respect to literature and conserved motifs. In addition, the pair shows high expression correlation between them. We use this additional information for detailed case studies by considering those pairs as high confident among all our predictions (see subsection 4 in Results). See Additional File 2 for better visibility of this figure and Additional File 3 for a list of TF-gene pairs together with Pearson coefficients. Generated by Cytoscape [36] and yED graph editor [37].

In contrast to earlier works on identification of transcriptional modules themselves, we seek to identify only the most reliable TF-gene functional links from those modules. To this end, we first identify coherent modules from PTMs using expression and functional annotation data (Step2 in Figure 1; see Methods). We have two p-value threshold parameters to define coherent modules: one for expression coherence (***τ***_***e***_) and the other for function coherence (***τ***_***f***_). Given two threshold parameters, we predict regulatory TF-gene pairs from functional intersection of identified coherent modules (see Methods). We varied the parameters by taking all combinations of four significant thresholds: 0.001, 0.005, 0.01, and 0.05. Then, positive predictive values (PPVs) were calculated with respect to the combined reference of the literature and conserved motif references (a total of 3962 TF-gene pairs; see Methods). In this work, we report all results based on ***τ***_***e ***_= 0.005 and ***τ***_***f ***_= 0.05, which gives the highest PPV among the 16 combinations (see Additional file 1). With this combination of p-value thresholds, we obtained 89 coherent modules with a total of 47 coherent functional categories (out of the total 557 modules tested). 20 out of the 47 enriched functions are shared by at least two of 42 coherent modules with common target genes, i.e., coherent linker genes. This functional intersection resulted in 66 coherent linker genes and 18 associated TFs, yielding 177 TF-gene functional pairs as represented in Figure 2 (see Additional file 2 for better visibility). Notice that coherent modules themselves are not the focus of our analysis.

### Evaluation

#### Validation

We first confirmed the validity of our method by assessing our predicted TF-gene functional pairs in view of the ChIP-chip data we started with (removing all uncharacterized genes for this validation; see Methods). We calculated the two performance measures, positive predictive value (PPV) and sensitivity (SNST), with respect to literature and conserved motif reference datasets (see Methods). As shown in Table 1, we obtained higher PPVs at the expense of lower SNSTs, which is to be expected as we aim at prioritization of regulatory links for individual experimental validation.

**Table 1:** Comparison of performance measures. For evaluation of our method, we compared our predicted TF-gene functional pairs (fourth column in bold, 'Our_final') with ChIP-chip results with annotated genes only (first column, 'ChIP-chip'), two prescription steps of our method (second and third columns, 'CM' and 'FI_TM'), and two other previous algorithms (fifth and sixth columns, 'GRAM' and 'MA-Networker'). Two performance measures were calculated, PPV and SNST for two reference datasets (see Methods). CM = all TF-gene pairs from coherent modules; FI_TM = TF-gene pairs from functional intersection among the initial putative transcriptional modules from ChIP-chip; lit = literature reference; con_mot = conserved motif reference; N_pairs = number of TF-gene pairs; N_genes = number of genes in the pairs; N_TFs = number of TFs in the pairs. Further comparison analysis is performed in Additional file 1. See the main text for details.

	ChIP-chip	CM	FI_TM	**Our_final**	GRAM	MA-Networker
PPV (%)(lit; con_mot)	4.6; 32.7	6.0; 35.8	18.2; 24.5	***13.6; 48***	6.3; 24.6	6.5; 38.6
SNST (%)(lit; con_mot)	13.7; 40.2	4.2; 10.5	1.7; 0.9	***2.0; 2.9***	7.9; 12.8	6.9; 16.8
N_pairs	3598	857	110	**177**	1518	1272
N_genes	1837	393	44	**66**	655	989
N_TFs	95	24	30	**18**	69	36

We also investigated whether coherent modules themselves or functional intersection alone could have given us better performance than our combined strategy (see Methods). First, taking *all *pairs in coherent modules without functional intersection does not yield higher PPVs at the expense of SNSTs for both reference sets (column 'CM' in Table 1), indicating that functional intersection is an important step. Second, we took TF-gene pairs from functional intersection of the initial PTMs from the ChIP-chip data (584 modules in total) without applying the expression coherence test. This functional intersection from the initial PTMs yields higher PPV than our predictions (18.2% vs. 13.6%) for the literature reference but lower PPV than ours (24.5% vs. 48%) for the conserved motif reference (column 'FI_TM' in Table 1). This suggests that functional intersection is the key to good performance with respect to literature. However, using conserved motifs as a reference, the PPV (24.5% after functional intersection) is lower than the PPVs from either the ChIP-chip results alone (32.7%) or coherent modules above (35.8%) (see Table 1). On the other hand, the SNSTs after this functional intersection are lower than our predictions for both reference sets (Table 1). Therefore, the combination of both prescriptions is important for detecting regulatory signals from ChIP-chip data. Further support for validation of our method is presented in Additional file 1.

#### Comparison with other methods

The performance of our validated method was compared with results from two previous algorithms, GRAM [1] and MA-Networker [9], using the literature and conserved motif references. Both studies used the same ChIP-chip data by Lee et al. and different expression datasets which they combined from diverse publications (see Methods). We used the published results in their original papers for comparison. The GRAM algorithm predicted 1518 TF-gene pairs (in rich media condition) and the MA-Networker 1272 pairs. 66 pairs from GRAM and 67 pairs from MA-Networker overlap with our 177 pairs and 39 pairs were predicted by all the three algorithms (see Additional file 3; 469 pairs overlap in GRAM and MA-Networker). We observe that our method has higher PPV than the two methods and lower in SNSTs for both reference sets (columns 'GRAM' and 'MA-Networker' in Table 1). On the other hand, the two overlaps of 66 pairs and 67 pairs with the two algorithms give rise to yet higher PPVs with respect to the literature reference: 27% and 18% respectively. For the conserved motif reference case, the overlaps yield 50% and 45% PPVs respectively, which are similar to our performance of 48%. Of the 39 pairs predicted by all three algorithms, 10 pairs are found in the literature reference and 16 pairs in the conserved motif reference (25% and 41% PPVs respectively).

To illustrate the generic applicability of our approach, which does not depend on our definition of modules, we applied the functional intersection to the 106 final modules of the GRAM algorithm. This may be considered as analogous to our expression coherent modules in the absence of incorporation of functional annotation data. Then, PPVs were calculated and compared with those of their final modules for the two reference sets. The functional intersection yielded 23 pairs between 13 TFs and 9 genes (i.e., coherent linker genes) with higher PPVs than their own modules; 43.5% and 30.4% for the literature and conserved motif reference sets respectively (as compared with 6.3% and 24.6% in row 'PPV' and column 'GRAM' in Table 1). This illustrates that our approach of functional intersection may be applied to any set of modules identified in other works to yield more reliable regulatory links.

Finally, we present results of an additional comparison with a recent module prediction study by Lemmens et al. [7]. Their work integrated three types of data sources (ChIP-chip, gene expression, and conserved motifs), rather than two as in GRAM and MA-Networker. By applying our method to the same ChIP-chip and gene expression data [14,16] as in their study, we predicted 108 regulatory interactions and yielded 14.8% PPV with respect to the literature reference. For a comparison, we used their "seed modules" which contain 134 TF-gene interactions, a comparable number of predictions to ours. Their 134 predictions yielded 12.9% PPV with respect to the literature reference we used. Although the prediction accuracies are similar, there is only little overlap between the predicted sets of regulatory interactions (9 interactions in common, 3 of them are found in the literature reference). See Additional file 1 for more details on this comparison with Lemmens et al.

### Examples

We now continue with detailed inspections of some of our systematic results shown in Figure 2. It is well-known that activity profiles of TF proteins are not necessarily reflected in expression profiles of the corresponding genes because of post-transcriptional and post-translational regulations of TFs [17]. We took, however, any such correlation as an additional indicator of a functional relationship among our predictions and aimed at identifying all TF-target pairs with high correlation for detailed analysis. To this end, we calculated Pearson coefficients for our predicted TF-gene pairs and compared them with a background distribution of Pearson coefficients for all pairs between ~200 TFs of Harbison et al. [16] and all other genes. By taking those observed pairs whose coefficients fall within 5% of both tails from the distribution of all the coefficients (the two thresholds being 0.661 and -0.628), we obtained a list of 46 highly correlated pairs between 13 TFs and 27 target genes: 33 positively and 13 negatively correlated pairs (Figure 2). In the following we restricted ourselves to some of these more specific TF-gene pairs.

#### Functionally interacting proteins

As an application from our functional TF-gene predictions, the 46 pairs with high expression correlation can provide a basis for identifying functional interactions of proteins. We hypothesize that those target genes regulated by the same TF(s) with high expression correlation have related roles in more specific biological processes than those encapsulated by the 3rd level MIPS category. In Figure 2, we observe that some groups of genes are highly correlated with their common TFs. They include known examples such as the associations between Hir2 and the six histone genes [18], and the known role of Ino4 in the regulation of FAS1 and FAS2 [19].

As another such group of genes, our method yielded a group of 5 genes, KRE6, EXG1, SCW4, PSA1 and HXK2, which are highly correlated with their common regulator Swi6 (Figure 2). All these genes share a high-level annotation of 'C-compound and carbohydrate metabolism'. There is no literature evidence for the transcriptional regulation by Swi6, but all genes were found to have binding sites of Swi6 conserved in at least one other yeast species [16]. Previous experimental studies show that 4 out of the 5 gene products, Kre6, Exg1, Scw4 and Psa1, are related to the cell wall synthesis and that cell wall genes are controlled by cell cycle progression where Swi6 has a regulatory role [20,21]. The 4 proteins are specifically implicated in synthesis of either glucose chains (glucans) or mannose-bound proteins (mannoproteins) which are two main inter-connected components of the cell wall.

The remaining protein, Hxk2 (hexokinase 2), is known to be a major upstream regulator of the glucose signalling pathway, which also impedes on cell wall genes. Specifically, a glucan synthase subunit, Gsc2, is regulated by Hxk2 via Snf1 and Mig1 [20,22]. Hence, it is possible that Hxk2 is functionally related to the 4 other gene products through glucose regulation and utilization for glucan synthesis. Glucose signalling is also known to act downstream on the cell-cycle, although the precise mechanisms are not yet fully understood [23]. Our result may suggest a possible feedback onto glucose regulation through the regulatory interaction of Swi6 with HXK2.

#### Conserved binding sites for three regulators of CIS3

We predicted two target genes CIS3 and UTH1 regulated by three TFs, Swi6, Fkh2 and Ndd1. The expression profile of CIS3 (glycoprotein-encoding gene in cell wall) is highly correlated with all those three TFs (Pearson coefficients are 0.856, 0.801 and 0.765, respectively), which additionally supports functional regulation of the gene by the three TFs. On the other hand, UTH1 is not well correlated with the TFs (Pearson coefficients are between -0.1 and 0.3), hence we do not postulate a functional interaction between CIS3 and UTH1, in contrast to the analysis in the previous subsection. While conserved binding sites for all the three TFs were found upstream of UTH1, Harbison et al. [16] did not identify any conserved binding sites upstream of CIS3.

**Figure 3:**
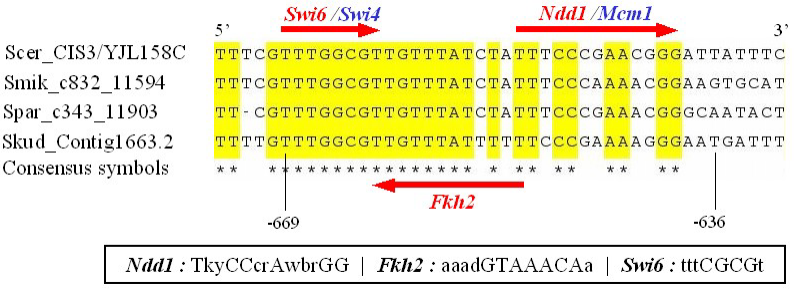
**Alignment of 4 orthologous 1 kb promoter regions for CIS3 and TF binding sites**. We show only the region (-636 to -669 upstream of the TSS) which contains conserved binding sites for all predicted regulators (Swi6, Fkh2 and Ndd1). We used the six-species alignment from SGD (including *S. mikatae*, *S. paradoxus *and *S. kudriavzevii*), but removed from the SGD output *S. castellii *which is more distant, and *S. bayanus *which has a small intergenic region of only 30 nucleotides. Consensus motifs of the TFs from Harbison et al. are shown in the box using the IUPAC code. We denote degenerate binding sites of Swi4 and Mcm1 in blue. See the main text for more details.

As we predicted that the three TFs functionally regulate CIS3, we searched for any putative binding sites of those TFs and their conservation across species in the upstream region of the gene. To this end, we used the matrices for Swi6, Ndd1 and Fkh2 provided by Harbison et al. [16] and scanned the 1 kb upstream region of CIS3 for matrix hits above the balanced thresholds introduced by Rahmann et al. [24]. We set the GC content of the background model to 50%. All putative binding sites detected are located within 34 base pairs (Figure 3). For the investigation of conservation of the putative TFBS region, we used the fungal sequence alignment tool in SGD [25] and found a high degree of conservation for 4 orthologous upstream regions (Figure 3).

It is worth noting that Ndd1-Fkh2 interactions have been suggested to be important in regulating G2/M-specific genes in cell cycle together with the MADS box protein, Mcm1, forming a permanent protein-DNA complex [26]. In fact, the position specific frequency matrix of Ndd1 from the study of Harbison et al. is very similar to that of Mcm1, so we were able to detect a binding site overlapping with that of Mcm1 (Figure 3). This indicates that Ndd1 could act as a functional co-factor, which cannot be distinguished from Mcm1 in ChIP-chip assays and motif scans. Similarly, Swi6 is known to have a regulatory function forming SBF or MBF complexes with Swi4 or Mbp1 respectively [27]. We found a binding site of Swi4 overlapping with the binding site of Swi6 (Figure 3). As before, it may not be possible to differentiate between the binding properties of these two factors. These inspections show that our method correctly predicted TFs which have a regulatory function among the components of the TF complexes, even though the regulatory relationship may be indirect. Taken together, this detailed investigation highly supports our prediction of the functional regulatory links between CIS3 and the three TFs.

## Discussion

Modular organization has been proposed as a fundamental principle in cellular systems and many computational algorithms have been developed to identify such modules. In particular, transcriptional modules have been extensively investigated using genome-wide data sources in yeast. It is practically impossible to verify all regulatory links identified in those modules in a single laboratory. As an attempt to overcome this problem, we developed a simple 3-step method to prioritize regulatory links in modules using three types of data sources (Figure 1). First, we defined putative transcriptional modules based on genome-wide binding data from ChIP-chip. We then identified *coherent modules *using gene expression data and functional annotation data. Finally, and in contrast to other works, we focused on *coherent linker genes *which appear in several coherent modules by way of *functional intersection*. These genes and their regulators from coherent modules resulted in a list of 177 regulatory interactions which have a high level of support and serve as reliable candidates for further experimental validation.

Our analysis showed that the proposed approach increased the positive predictive value (PPV) when compared with two previously published results by Bar-Joseph et al. and Gao et al., at the expense of sensitivity. This should be expected considering the fact that we integrated one additional data source of functional annotation with the two data sources of ChIP-chip and gene expression which the other two algorithms used for their predictions. One point to make, however, is that while we utilized functional annotation data for the purpose of prediction, they used annotation data for validation of their prediction. Note also that because the validation using annotation data involves over-representation or enrichment of genes in sets of genes, it cannot serve for validation of *all *predicted functional target genes. In addition, while those works utilized gene expression data to derive coherent modules from ChIP-chip binding data, their published work did not focus on individual regulatory interactions. Their predicted interactions are simply all members of statistically predicted modules themselves. In contrast, our predictions do not exclusively aim at modules, but individual regulatory interactions, which we obtained by means of functional intersection. By this prioritization approach we purposefully predicted less functional associations (less sensitivity), but doubled PPV with respect to the literature reference (13.6% vs. 6–7%). Although this validates our approach, it may illustrate a limitation of the literature reference which covers only a fraction of all experimentally verified genes to date. Because of this limitation we also compared the different methods with respect to a more comprehensive reference set of predicted regulatory interactions. These predicted interactions are based on updated ChIP-chip data and sequence conservation across other yeast species. We took them as an indication for functional interactions. Using this reference set, we achieved 48% as compared to 25–39% from the two other works. We stress that all methods compared here have their own specific aims and merits although they share the overall goal to derive functional interactions from physical interactions (as provided by ChIP-chip). A conservative strategy to identify functional interactions, would combine the results from multiple algorithms. We provide our results of such analysis in Additional file 1.

Using the updated ChIP-chip dataset by Harbison et al. [16] also enabled us to compare our approach with a recent algorithm (ReMoDiscovery) by Lemmens et al. [7]. Their algorithm is similar in spirit to the GRAM algorithm but integrates three types of data sources (ChIP-chip, gene expression, and conserved motifs) in a concurrent way. This is different from our sequential approach, but yielded similar prediction accuracy. Yet, in terms of predicted regulatory interactions, there was only little overlap with our results, indicating the complementarity of these two methods.

In general, it is difficult to directly compare the performance of different algorithms which are designed for different purposes. Our comparison of published results highlights the fact that different approaches have so far been used with different aims and yield different trade-offs between specificities and sensitivities. A more comprehensive evaluation study would require re-running different algorithms in different regions of parameter space. Notice though that in this work we did not vary p-value thresholds of ChIP-chip results to adjust PPV or sensitivity as was done by Bar-Joseph et al. [1], for instance.

Another strategy to improve all methods is to incorporate activity profiles of TFs (i.e., protein concentrations). Experimental data of protein concentrations are currently lacking and using mRNA expression profiles of TF-encoding genes is not promising because of weak correlation as we discussed earlier [17]. Also notice that the computational effort to infer activity profiles of TFs, as attempted by the MA-Networker algorithm, did not result in better performance than our simpler approach. Here we focus on TF-gene pairs with high expression correlation (coloured edges in Fig. 2) only for the purpose of a detailed analysis, but not as part of the systematic study.

Specifically, we suggested 5 functionally interacting proteins in cell wall formation and a possible feedback regulation of glucose utilization through Swi6. We also predicted that the glycoprotein-encoding gene CIS3 is regulated by three cell-cycle regulators, Swi6, Ndd1 and Fkh2. In addition, our detailed analysis revealed that the conserved binding sites of the three factors are located very close to each other. We further identified binding sites of Swi4 and Mcm1 which overlap with those of Swi6 and Ndd1 respectively, suggesting formation of two complexes, Swi6-Swi4 (SBF) and Ndd1-Fkh2-Mcm1. The two complexes may interact with each other through Fkh2 on the basis of the identified binding sites. On the other hand, a previous study on cell cycle by the Young laboratory identified CIS3 as a target of two cell cycle activators, the SBF complex and Fkh2, but not as a target of the Ndd1-Fkh2-Mcm1 complex. See Table 1 in [28]. Hence, our results suggest a new regulatory link between the Ndd1-Fkh2-Mcm1 complex and CIS3. It might also be the case that Fkh2 recruits either the SBF complex or the two other components of the Ndd1-Fkh2-Mcm1 complex according to distinct cell-cycle phases.

By the design of functional intersection, our predictions suggest multiple transcription factors for each gene. This could be taken as a sign of combinatorial regulation. We would like to caution, however, that the inference of combinatorial regulation requires further analysis, such as the vicinity of binding sites, as in the example of CIS3, or a clear indication that the combination of factors is required for synergistic expression [29]. Our predicted list of multiple transcription factors did not result from such an analysis since we did not pursue the issue in this work.

## Conclusion

Here we proposed a simple method to obtain functional regulatory interactions from physical interactions (e.g. from ChIP-chip data). To this end, we utilized gene expression and functional annotation data which helped to refine transcriptional modules and identify coherent linker genes for prioritization. We demonstrated that our method is able to increase the fraction of functional interactions with respect to two reference datasets and complementary to other existing methods. Finally, we suggested several novel individual interactions for further mechanistic analysis and experimental validation.

## Methods

### Data sources

#### ChIP-chip data

We used genome-wide ChIP-chip data of the laboratory of Richard Young [13]. They experimented 106 TFs in rich media conditions. We used a binding p-value threshold 0.001 as suggested in their paper to define putative functional target genes. They provided a data matrix where each intergenic region assayed is assigned to a downstream neighbouring gene. From this matrix, a set of potential target genes for each of the 106 TFs can be identified with the p-value threshold. Although this original dataset has been supplemented by new data with more TFs and conditions [16], we apply our method to the older data to compare our results with other methods which also used the same data of Lee et al. [13].

#### Gene expression data

It is biologically important to have independent experimental datasets in which cellular conditions are comparable if one tries to integrate them for analysis. Therefore, we selected gene expression data in view of the experimental conditions of the ChIP-chip data we used in this study. Only gene expression experiments were extensively done in many diverse conditions on a genome-wide scale. ChIP-chip experiments by Lee et al. [13] were conducted under normal growth conditions in rich media and so we focused on elutriation conditions (size-based synchronization) in expression data by Spellman et al. (1998). Two other methods they used for the synchronization of cell cycle were involved with alpha-factor pheromone treatment and temperature-sensitive cdc15 mutation, which introduced characteristic artifacts of mating and heat shock respectively [14]. Those artefacts are not expected in the conditions of the ChIP-chip assays. Therefore, we used the elutriation data as the experiment was not involved with such artifacts. The data consist of 14 time points taken every 30 minutes for 6.5 hours.

#### Functional annotation data

We used the functional categories provided by the Munich Information Center for Protein Sequences [MIPS, [15]] upto the 3rd level of the category hierarchy (classification version 2.0). More detailed annotations were pruned at the 3rd level, resulting in about 200 categories examined in total. They contain upto ~750 proteins with an average of 56, excluding the category, 'unclassified proteins', which contains about 2000 proteins.

### Reference datasets

#### Literature collection

The first reference set we used is 1207 TF-gene pairs compiled from three literature-curation sources: (1) Lee et al.'s curation of 1049 pairs excluding computational regulatory motif results [13] (2) TRANSFAC database for 342 pairs [[30], version 10.4] (3) Siddharthan et al.'s curation of 72 pairs [31]. Notice that the reference data may contain TF-gene pairs where TFs act as mere DNA-binding factors rather than functional regulators.

#### Conserved motifs

The laboratory of Richard Young recently advanced their ChIP-chip technology and applied it to yeast with 203 TFs [16] (compare with 106 TFs in Lee et al. [13]). Based on their binding data and sequence data from four yeast species, they identified conserved binding motifs for 102 TFs using a variety of motif detection algorithms (this was not done in the work of Lee et al. [13]). It is widely believed that conserved motifs across species indicate their functional roles [32-34]. While the 'phylogenetic footprinting' approach will introduce errors, it provides a more comprehensive picture of regulatory links than manual curation of literature. Hence, we take the dataset of conserved motifs as a second reference set independently of the literature-based reference. We compiled 2922 TF-gene pairs from the motif analysis results of Harbison et al. [16] maintained in the *Saccharomyces *Genome Database (SGD) [25]. The list of 2922 pairs is derived from predicted binding sites which are conserved in at least two *Saccharomyces *species, other than *S. cerevisiae*. Note that this set contains more than twice as many predicted interactions as the literature reference set.

### Main procedure to predict regulatory TF-gene interactions

#### Identification of putative transcriptional modules from ChIP-chip data

##### Enumeration of large bicliques

Regulatory interactions between transcription factors (TFs) and target genes can be represented as a bipartite graph, with edges going from a set of TFs to a set of target genes. A biclique K is a bipartite graph such that an edge is realized from every vertex of a TF set (***F***) to every vertex of a gene set (***G***), i.e.,

(1) *K *= (*F *+ *G*, *E*),

where ***E ***is a set of all possible edges from ***F ***to ***G***. (i.e., ||***E***|| = ||***F***|| * ||***G***||). Input to our method is a set of bicliques. Our data typically are quite sparse, i.e., the number of edges in a bipartite graph is much smaller than the size of the entire TF set multiplied by the size of the entire gene set. For example, in the case of the ChIP-chip data introduced above, a p-value threshold of 0.001 results in a total of 4611 regulatory interactions and 584 bicliques generated by our program described below. Generally, a bipartite graph will contain a large number of bicliques. We have implemented a simple enumeration algorithm for large bicliques with the constraint that ||G|| >= 5 (for the purpose of statistical assessment in our subsequent analysis).

Let the set F of all factors be ordered. In the first pass of our program, each factor is inspected whether it is connected to 5 or more genes. These constitute the first set of (trivial) bicliques. The idea is then to extend those bicliques to find the bicliques with 2 factors, then with 3 factors, etc. Now assume that a set of all bicliques with m factors has been determined. The algorithm then runs iteratively through all the bicliques with m factors and adds an additional factor from the ordered list of factors to each biclique, if that factor targets 5 or more genes from the set of genes in the biclique in question. Thereby we obtain a new biclique with m+1 factors. The gene set of this new biclique is the intersection of the gene set in the old biclique and the set of target genes of the newly introduced factor. Since this procedure observes the order of factors, bicliques are not discovered repeatedly. However, at each step the algorithm may generate a new biclique with an identical set of genes already contained in the old biclique, in which case we discard the old one. Notice that this prescription may still result in bicliques with the same set of genes after the whole iteration has finished.

##### Putative transcriptional modules

Since our subsequent analysis will deal only with the gene sets induced by the bicliques derived above, we first merge those redundant bicliques which contain identical sets of genes, so as to avoid any computational overhead. The merged biclique is designed to have those transcription factors which belong to two or more of the redundant bicliques (i.e., TFs with multiplicity >= 2). In this way, we generated 584 non-redundant bicliques from the ChIP-chip data by Lee et al. [13], the maximum number of TFs in a biclique being 7. We also call them putative transcriptional modules (PTMs), and they are the input to our subsequent analysis of coherent modules (see Step 1 in Figure 1).

#### Identification of coherent modules

##### Expression coherence

Given a transcriptional module and an expression dataset we calculate Pearson correlation coefficients, *r*, for all pairs of expression profiles of target genes in the module and take the average of the absolute values of the coefficients. The reason why we take the absolute value is that we consider both positive and negative correlations as the signals for possible co-regulation. We define this average value, ***ξ***, in general as follows,

(2) $$\begin{array}{cc} \zeta \equiv \frac{1}{L}\sum_{k=1}^{L}\left|r_{k}\right|, & L=\left(\begin{array}{c} N\\ 2 \end{array}\right), \end{array}$$

where ***L ***is the number of all pairs of ***N ***target genes in each module and ***r***_***k ***_is the Pearson coefficient for a pair ***k***. We take ***ξ ***as a statistic for the significance test of expression coherence. For background ***ξ ***values, we generated random modules by sampling the same number of genes as the module in question. We estimated a p-value of expression coherence, ***p***_***e***_, for each observed module by the fraction of the number of those random ***ξ***'*s *that are equal to or greater than the observed ***ξ ***with respect to the number (***K***) of randomly sampled groups, which is ***K ***= 1,000 in this study,

(3) $$p_{e}=\frac{\left\|\left\{\zeta_{k}|\zeta_{k}\geq \zeta ,k=1,2,...,K\right\}\right\|}{K},$$

where ***k ***is an index for random modules. Transcriptional modules with p-values less than a threshold, ***τ***_***e***_, are deemed expression coherent modules.

##### Function coherence

Given a transcriptional module and a functional category (from MIPS), we assess enrichment of the functional category among target genes using the standard method [35]. Function coherence is meant to be the same as enrichment of a functional category throughout this study. The assessment of function coherence for each category was done by calculating a hypergeometric p-value which is defined as follows,

(4) $$p_{f}=1-\sum_{k=0}^{K_{f}-1}\frac{\left(\begin{array}{c} M_{f}\\ k \end{array}\right)\left(\begin{array}{c} N-M_{f}\\ S-k \end{array}\right)}{\left(\begin{array}{c} N\\ S \end{array}\right)},$$

where ***f ***is a functional category, ***N ***is the number of all genes which are annotated to at least one functional category, ***M***_***f ***_is the number of all genes which are annotated to the functional category ***f***, ***S ***is the number of all target genes in a module of interest, and ***K***_***f ***_is the number of target genes in the concerned module which are annotated to the given functional category ***f***. A functional category for each module is deemed coherent if ***p***_***f ***_is less than a prescribed threshold (***τ***_***f***_). Note that we may obtain multiple coherent functions in each module. We do not correct p-values for multiple testing. For each module, ***p***_***f ***_is defined for only those categories in which ***K***_***f ***_is greater than 0.

##### Coherent modules (CMs)

A transcriptional module is called a coherent module (CM) if both p-values, ***p***_***e ***_and ***p***_***f***_, are less than the two thresholds, ***τ***_***e ***_and ***τ***_***f***_, for expression and function coherence test respectively (Step2 in Figure 1), i.e.,

(5) *CM *= {*TM *= (*F*, *G*) | *p*_*e *_<*τ*_*e *_and *p*_*f *_<*τ*_*f*_}.

#### Identification of coherent linker genes

For a given list of coherent modules (CMs), we further focus on those functional categories which are coherent in *multiple *CMs. For a particular coherent function, we identify all CMs which *share *that function. Then, we identify *common *target genes in those CMs which are annotated to that function. We refer to this identification step as "functional intersection". Those filtered genes are called "coherent linker genes" as they link CMs. It should be noted that we require those coherent linker genes to appear in *all *those CMs. In other words, they are claimed to possess the strongest functional signal among others in CMs. Regulation of coherent linker genes by associated TFs in corresponding CMs constitutes our prediction of functional TF-gene pairs (Step 3 in Figure 1).

### Evaluation of the method

As performance measures, we calculated (1) positive predictive value (PPV) and (2) sensitivity (SNST) which are defined as the number of true positives (predicted TF-gene individual pairs that are found in a reference set) divided by (1) the number of predicted TF-gene pairs (2) the number of all reference TF-gene pairs. Notice that true negatives cannot be defined because there is no reference for the *absence *of regulatory relationships between TFs and genes. We used the above-mentioned two reference datasets for evaluation.

#### Validation

For the purpose of validation of our method, we compared the performance measures from our predicted TF-gene pairs and the original ChIP-chip data we used at a binding p-value threshold of 0.001 (4611 TF-gene pairs between 96 TFs and 2326 genes). We removed all uncharacterized genes from the ChIP-chip results for the validation to avoid a possible bias of our method towards annotated genes resulting from MIPS function data we incorporated. This leaves us with 3598 TF-gene pairs between 95 TFs and 1837 genes (Table 1). In addition, we validated each of the two steps in our strategy separately, (1) identification of coherent modules and (2) functional intersection among modules. The two performance measures were calculated and compared with our predictions from the combined strategy by taking (1) *all *TF-gene pairs from coherent modules *themselves *and (2) TF-gene pairs from functional intersection among *PTMs*, respectively.

#### Comparison

We compared our predicted TF-gene pairs with those of the previous two algorithms: GRAM [1] and MA-Networker [9], using the results provided in their original papers. Bar-Joseph et al. used the same ChIP-chip data along with a compiled expression dataset (over 500 conditions) to produce clusters of genes and regulators. We took TF-gene pairs in their final 106 clusters in rich media conditions. Gao et al. also used the same ChIP-chip data along with a compiled expression dataset (over 700 conditions). Their algorithm aimed to identify functional and non-functional target genes based on TF activity profiles they inferred using a multivariate regression model. We used the results of functional target genes and their TFs for comparison.

## Authors' contributions

RB, TM and MV conceived the study about coherent transcriptional modules and HFL developed the idea and conceived the study about prioritization. HFL collected the data, developed the methodology, and produced the results with comments from TM. The manuscript was written by HFL and TM and critically reviewed by RB and MV. The authors approved the final version of the manuscript.

## Supplementary Material

Additional file 1 Supplementary material. This file contains supplementary material for the main text. Click here for file

Additional file 2 Figure 2. This file contains the same Figure 2 in the main text for better visibility. Click here for file

Additional file 3 Final prediction list. This text file contains our final prediction of 177 TF-gene interactions in Figure 2 of the main text along with other informations as described in the header of the file. Click here for file
