# Supplementary material for: Prioritization of gene regulatory interactions from large-scale modules in yeast
Source: BMC Bioinformatics. 2008 Jan 22;9:32. doi: 10.1186/1471-2105-9-32 (PMC2244593; doi:10.1186/1471-2105-9-32)
Supplement: Additional file 1 — Supplementary material. This file contains supplementary material for the main text. [file 1471-2105-9-32-S1.pdf]

Supplementary Material

For

Prioritization of gene regulatory interactions from large-scale modules in yeast

Ho-Joon Lee[1], Thomas Manke[1] , Ricardo Bringas[2] and Martin Vingron[1]

[1] *Max Planck Institute for Molecular Genetics, 14195 Berlin, Germany*

[2]*Centro de Ingenieria Genetica y Biotecnolgia, Cubanacan, CP 6162, La Habana, Cuba*

> E-mail addresses :

Ho-Joon Lee,	lee@molgen.mpg.de
Thomas Manke,	manke@molgen.mpg.de
Ricardo Bringas,	ricardo.bringas@cigb.edu.cu
Martin Vingron,	vingron@molgen.mpg.de

CONTENTS

1. Parameter selection for coherent modules

2. Further validation of our algorithm

3. Overlaps with GRAM and MA-Networker

4. Analysis of ChIP-chip data by Harbison et al.

5. References

1. Parameter selection for coherent modules

We have two p-value threshold parameters to identify coherent modules in our method : one for expression coherence (τ_e) and the other for functional coherence (τ_f). Final predictions are made once coherent modules are identified with a specific combination of the two parameters (see subsection of 'Identification of coherent modules' in Methods section in the main text). We varied the two parameters by taking all 16 combinations of four different statistically significant values : 0.001, 0.005, 0.01, and 0.05 (Table S1). Then, a positive predictive value (PPV) was calculated for final predictions from each combination. For our parameter selection, PPVs were calculated with respect to the combined reference of the literature and conserved motif references (a total of 3962 TF-gene pairs; see Methods in the main text), rather than to calculate them for each of the references as in the main text (where we aimed at more careful analysis). The choice of the two parameter values, τ_e = 0.005 and τ_f = 0.05, in the main text was made on the basis of the highest PPV among the 16 combinations (the grey cell in Table S1). All those combinations gave rise to at least 100 TF-gene pairs. Note that increasing p-values does not necessarily provide a superset of or more predictions because functional intersection should be applied to *more* coherent modules dropping out some of coherent linker genes identified at lower p-value thresholds.

Table S1. Positive predictive values (PPVs) from 16 combinations of the two parameters, τ_e and τ_f

		τ_f			
		0.001	0.005	0.01	***0.05***
τ_e	0.001	36	38	39.9	44.8
	0.005	41.5	44.1	45.8	***53.1***
	0.01	40.2	43.1	45.1	51.1
	0.05	40.2	43.5	45.8	48.5

2. Further validation of our algorithm

We presented the validation results for our proposed algorithm in the main text (see subsection 3.1 in Results section). We further assessed our 3-step algorithm against several other alternative methods from the three steps, (1) expression coherence, (2) functional enrichment, and (3) module intersection, by calculating the two performance measures, PPV and SNST (see Methods in the main text). Here we present results based on the combined set of the two references as in Section 2 above. Similarly to the main text, we first confirm the validity of our algorithm by assessing our predicted 177 TF-gene pairs in view of the ChIP-chip results alone we started with. We get higher PPV and lower SNST as we expected ('ChIP_all' in Figure S1). To be more conservative, we removed all uncharacterized genes from the ChIP-chip results and the same conclusion was drawn ('ChIP_anno' in Figure S1).

We also validated our method by checking our prescription at each step. That is, we asked if each prescription is a contributing factor for better prediction power. First, all genes which appear at least twice (naïve linker genes or NLG) among expression coherent modules without functional enrichment do not yield higher PPV at the expense of SNST ('NLG_ECM' in Figure S1), neither do all TF-gene pairs in coherent modules ('All_CM' in Figure S1). This means that functional intersection is an important step. We also tested those functionally enriched genes which appear *at least twice* in coherent modules. These so-called coherent weak linkers (or CWLs) are different from coherent linker genes (CLGs) in the main text because CLGs should appear in *all* coherent modules with the same enriched functional category. As seen in Figure S2, CWLs yield less PPV as well, suggesting that CLGs show a stronger signal. In addition, even if we take either naïve or functional linker genes from *all initial* TMs without incorporating expression data, they give rise to less PPV too ('NLG_TM' and 'FLG_TM' in Figure S1).

In addition, we assessed significance of our increased PPV and SNST by randomly sampling TF-gene pairs from each alternative method as many as our predicted TF-gene pairs (177 pairs). A p-value is calculated as the fraction of 10,000 sets of 177 random TF-gene pairs which give rise to a performance measure equal to or larger than our proposed method. All p-values for both PPV and SNST are less than 0.05 (see Figure S2 for part of this significance test results). Therefore, we conclude that our proposed method is valid.

Figure S1. Comparison of performance measures for alternative methods

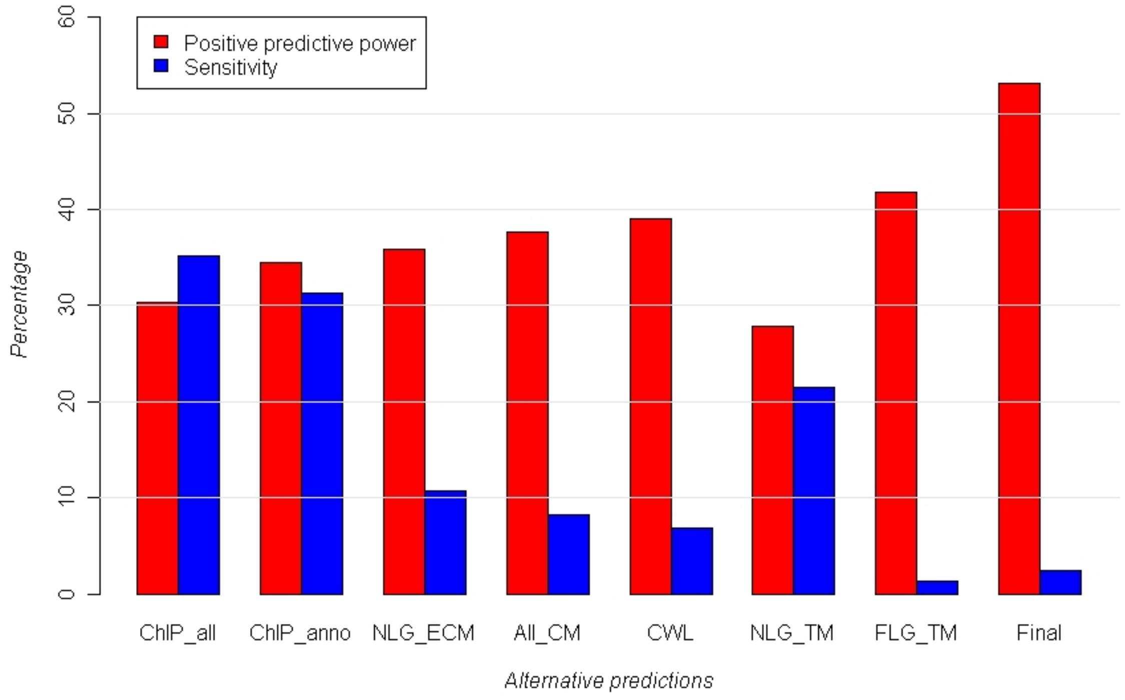

Figure S2. Significance of our performance. We show distributions of PPVs (upper panel) and SNSTs (lower panel) for random sets of 177 TF-gene pairs from some of the alternative methods in Figure S1. The legend indicates the following : 'ARP1', from ChIP-chip original results with annotated genes only ('ChIP_anno' in Figure S1); 'ARP2', from all pairs in CMs ('ALL_CM' in Figure S1); 'ARP3', from coherent weak linkers ('CWL' in Figure S1). 'ARP4' is based on coherent linker genes from random 102 ChIP-chip modules corresponding to our 102 expression coherent modules.

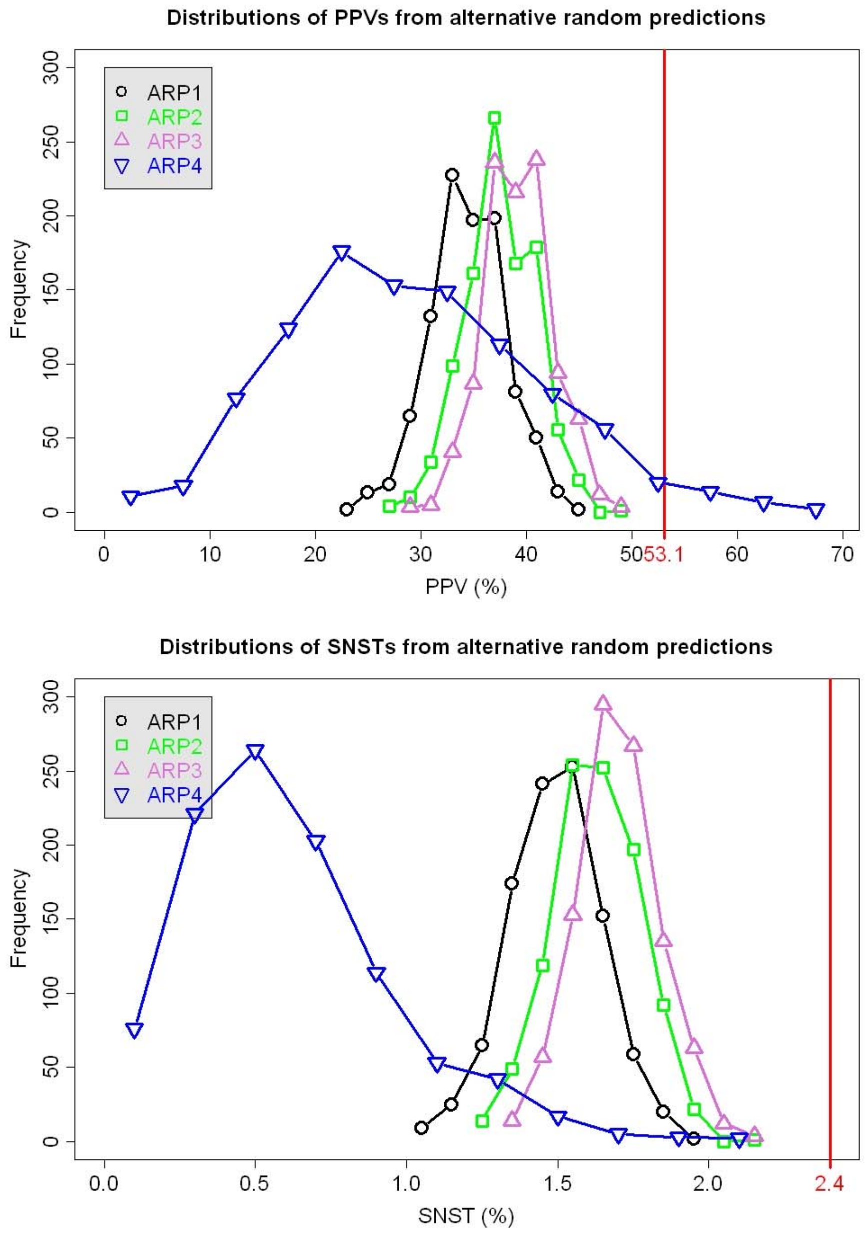

3. Overlaps with GRAM and MA-Networker

Here we investigate overlaps of our method with the two other methods we compared in the main text. In Table S2 (and Figure S3), we compared PPVs for unique and overlapping predictions of GRAM, MA-Networker, and our algorithms. In general, overlapping predictions are more reliable with respect to the literature reference compared with any unique predictions made by a single algorithm. This is not true for the case of overlapping predictions by all the three algorithms with respect to the conserved motif reference (41% in the red cell). Our total predictions and the overlap with GRAM yields better PPVs (the pink cells). In addition, our unique predictions achieve as good PPV for conserved motifs as the overlapping predictions (the green cells). On the other hand, the overlap of 469 pairs predicted by both GRAM and MA-Networker does not yield as good PPVs as our predictions (11.5% and 42% for the two references respectively; not shown in the table).

Table S2. Further comparison of prediction performances. We calculated PPVs for unique and overlapping predictions (TF-gene regulatory pairs) made by GRAM, MA-Networker, and our algorithms (named 'bar', 'gao', and 'our' respectively in the table). For two algorithms A and B, 'A \ B' in the table means the set of unique predictions made by A but not B and 'A & B' the intersection of predictions made by both A and B. 'our final' is the 177 final predictions from our algorithm and 'overlap of all' is the intersection of predictions made by all the three algorithms. 'PPV_lit' and 'PPV_motif' are PPVs with respect to the literature and conserved motif references respectively (see Methods in the main text). 'N_pairs' is the number of TF-gene predictions in each column. See Figure S3 for diagrams of this table.

	our final	our \ bar	bar \ our	our & bar	our \ gao	gao \ our	our & gao	overlap of all
PPV_lit (%)	13.6	5.4	5.3	***27.3***	10.9	5.9	***17.9***	***25.6***
PPV_motif (%)	***48***	***46.8***	23.5	**50**	***50***	38.3	*44.8*	*41*
N_pairs	177	111	1452	66	110	1205	67	39

Figure S3. Diagrams of prediction performances. The three diagrams represent Table S2 above. The pair of values in each parenthesis shows (PPV_lit, PPV_motif) of the corresponding column in Table S2.

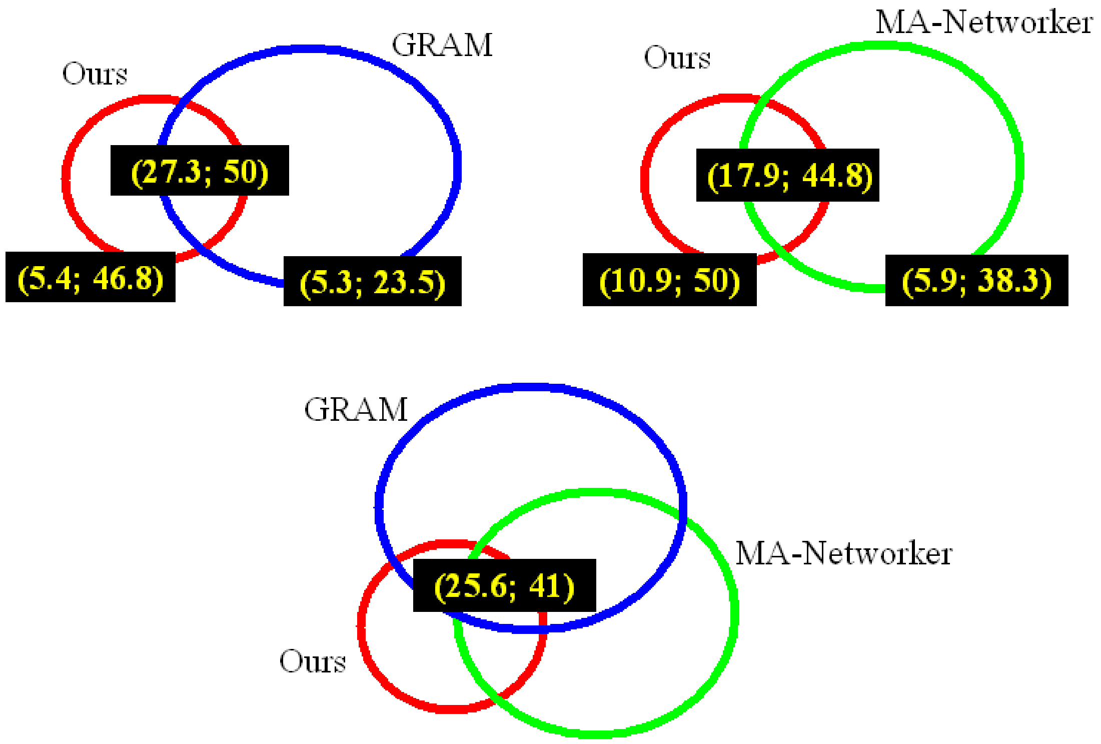

4. Analysis of ChIP-chip data by Harbison et al.

During revision of the manuscript, we applied our method to the larger ChIP-chip dataset by Harbison et al. (Harbison *et al.* 2004) to compare with the algorithm, ReMoDiscovery, (Lemmens *et al.* 2006) which used that dataset.

Lemmens et al. (Lemmens *et al.* 2006) recently developed a module discovery algorithm which integrates ChIP-chip, gene expression and, in contrast to our method, conserved motif datasets in a concurrent way. We took the 134 TF-gene pairs from their highly reliable seed modules, the number of which is similar to that of our predicted pairs (108; see below). When we tried their algorithm locally, we were not able to find stringent parameter values (5 parameters) which would give a comparable number of predictions. The 134 pairs yield PPV of 12.9% with respect to the literature reference. Here, we did not consider the conserved motifs from Harbison et al. as a reference as this would be circular.

With this new ChIP-chip dataset our method generated a total of 1989 modules (bicliques) with 5 or more target genes at a p-value threshold of 0.001 and applied to the same gene expression data by Spellman et al. as Lemmens et al. and the MIPS annotation dataset. As described in the main text, we varied our two parameter values of τ_e and τ_f to achieve the highest PPV of 14.8% at τ_e = 0.001 and τ_f = 0.005 with prediction of 108 TF-gene pairs. The increase of 1.9% of our method against 12.9% PPV by ReMoDiscovery may be insignificant considering the fact that we predicted 26 less pairs. The overlap between the 134 pairs of ReMoDiscovery and the 108 pairs of our method is 9 (3 of them are found in the literature reference). Note also that both 14.8% and 12.9% PPVs by the two methods are similar to 13.6% we achieved using the older ChIP-chip data by Lee et al. (Lee *et al.* 2002) in the main text.

Therefore, we conclude that (1) using the improved ChIP-chip data by Harbison et al. does not yield a significant increase in prediction precision with respect to the literature reference we used and that (2) both our method and ReMoDiscovery which utilize three types of data sources achieve similar precision but make different predictions of TF-gene regulatory interactions.

5. References

Harbison, C. T., D. B. Gordon, *et al.* (2004). **Transcriptional regulatory code of a eukaryotic genome**, *Nature* **431**(7004): 99-104.

Lee, T. I., N. J. Rinaldi, *et al.* (2002). **Transcriptional regulatory networks in Saccharomyces cerevisiae**, *Science* **298**(5594): 799-804.

Lemmens, K., T. Dhollander, *et al.* (2006). **Inferring transcriptional modules from ChIP-chip, motif and microarray data**, *Genome Biology* **7**(5): R37.
