# Supplementary figures and images for: Prioritization of gene regulatory interactions from large-scale modules in yeast
Source: BMC Bioinformatics. 2008 Jan 22;9:32. doi: 10.1186/1471-2105-9-32 (PMC2244593; doi:10.1186/1471-2105-9-32)

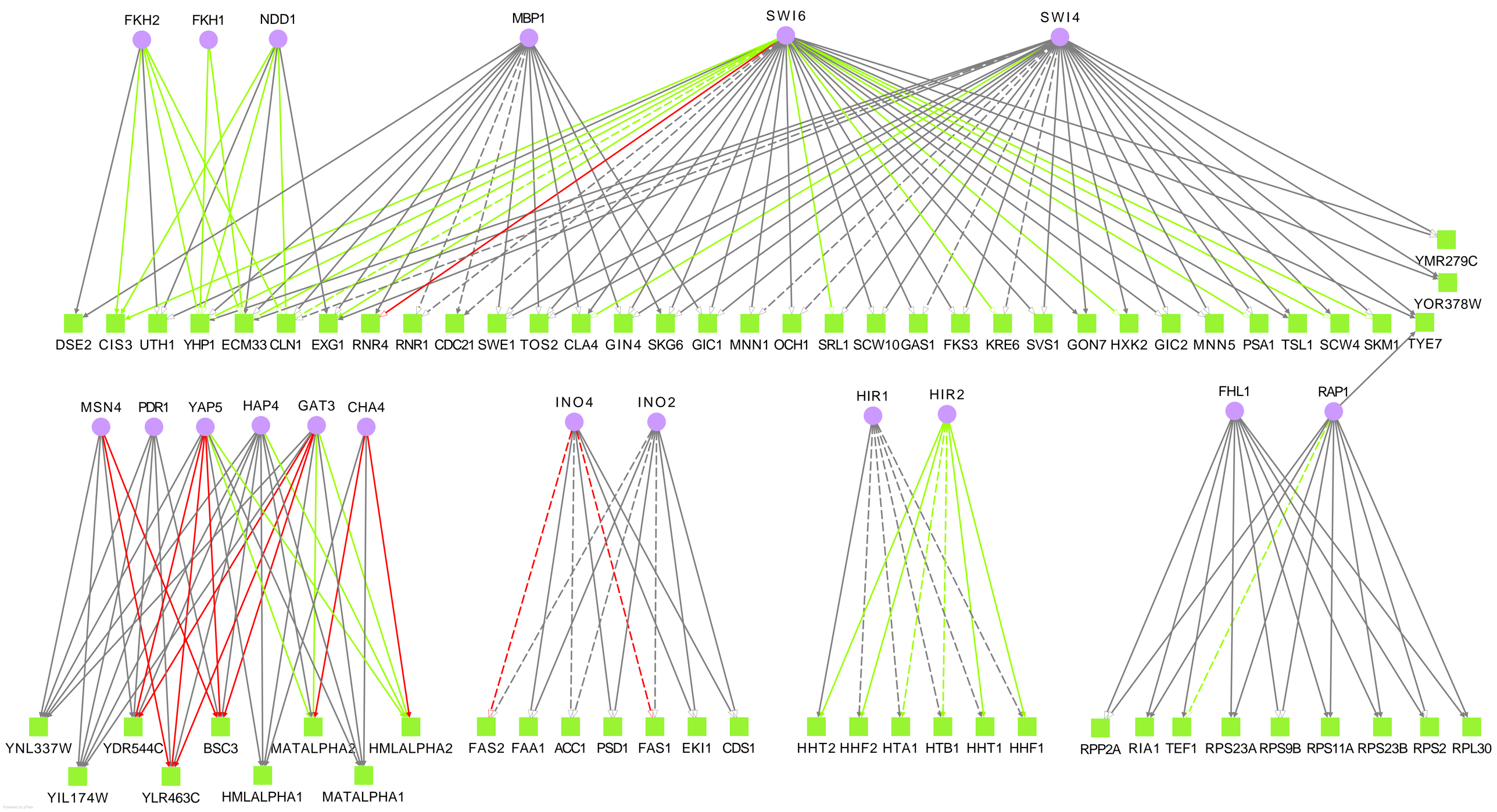

Supplement: Additional file 2 — Figure 2. This file contains the same Figure 2 in the main text for better visibility. [file 1471-2105-9-32-S2.pdf]
